# Intestinal parasitic infections among public and private schoolchildren of Kathmandu, Nepal: prevalence and associated risk factors

**DOI:** 10.1186/s13104-019-4225-0

**Published:** 2019-03-29

**Authors:** Jitendra Shrestha, Balkrishna Bhattachan, Ganesh Rai, Eun Young Park, Shiba Kumar Rai

**Affiliations:** 1 Shi-Gan International College of Science and Technology, Affiliated to Tribhuvan University, Kathmandu, Nepal; 2 College of Pharmacy, Mokpo National University, Mokpo, South Korea

**Keywords:** Intestinal parasites, *Giardia duodenalis*, Schoolchildren, Kathmandu, Nepal

## Abstract

**Objective:**

Intestinal parasitic infections (IPIs) are a major cause of morbidity among children in developing countries. Investigation about the etiological agents and socio-ecological pattern of the infection would help to design better preventive strategy. The previous studies reported high prevalence of IPIs among schoolchildren of Nepal. Though these data may be essential for the policymakers and researchers, in Kathmandu, the capital of Nepal it remains unexplored whether the types of school and socioeconomic status affect the IPIs or not. The present study is an extension of previous works to investigate causative agents and associated risk factors. We examined 508 stool samples of schoolchildren from two schools by formal-ether concentration technique and analyzed the data based on school types.

**Results:**

The overall IPIs rate was 19.9% (n = 101) with the dominance of protozoans (78.4%) over helminths (21.6%). *Giardia duodenalis* (32.7%) and *Ascaris lumbricoides* (21.8%) were the most commonly detected protozoan and helminth species respectively. Prevalence of IPIs was higher among children from public school (26.1%) than private school (12.1%). Higher infection rates were found among farmer’s children (29.0%) and Dalit children (36.2%). These findings reveal the different prevalence of IPIs among public and private schoolchildren and suggest the need of effective preventive measures.

## Introduction

Intestinal parasitic infections (IPIs) are one of the common cause of diarrhea in developing countries. Globally 1.5 billion people are infected with soil-transmitted helminths, above 267 million preschool children and over 568 million school-age children live in intestinal helminths prevalent area [1]. IPIs gradually exacerbate the nutritional status with an adverse effect on childhood development and increase morbidity among children [1]. In adults, IPIs it reduces work productivity and impairs the economic growth of developing nations [2]. In children, Insufficiency of safe drinking water, overcrowded population and poor personal hygiene with weak nutritional status have been identified as the risk factors for IPIs [3, 4].

Globally IPIs related morbidity has been considered as a major threat for public health. A prevalence of 85.7% has been reported in Ecuador for IPIs, with a majority of *Entamoeba histolytica/dispar* (57.1%) and *Ascaris lumbricoides* (35.5%) infections [5]. Similarly, a prevalence rate higher than 50.0% has been reported for soil-transmitted helminths in the northern part of India with a dominance of *A. lumbricoides* [6]. Moreover, IPIs were found prevalent and a cause of diarrhea among kindergarten and/or schoolchildren in Ghana [7] and China [8], respectively. In Nepal, a recent study [9] showed that the prevalence of IPIs decreased among schoolchildren from 61.0% in 1990 to approximately 20.0% in 2015, indicating a declining pattern in the last two decades. In Kathmandu, a study [10] reported that the prevalence of IPIs is higher among public schoolchildren than private schoolchildren. In this study, we aimed to assess the recent prevalence pattern of IPIs among public and private schoolchildren of Kathmandu as the extension of previous research.

The objectives of this study are to compare the prevalence rate of IPIs among private and public schoolchildren of Kathmandu and to evaluate possible associations of IPIs patterns with demographic, socio-economic and behavioral factors. Our findings would be beneficial to implement possible preventive measures to control and cure the parasite associated infections among the dwellers.

## Main text

### Methods

#### Study design and site

This cross-sectional type of study was carried out from March to September 2014. This study was done among public and private schoolchildren of Kathmandu, Nepal. Kathmandu Valley is situated at an average elevation of 1400 m (4600 ft) above the sea level and has an approximate population density of 4416 per km^2^ within a total area of 50.7 km^2^ [11].

#### Study population

Among many public and private schools in Kathmandu, a public and a private school were randomly selected with a similar location. The total student population was 1027 and 673 in public and the private school, respectively. The sample size (508) was calculated using Cochran’s sample size formula for categorical data [12] and a total of 508 stool samples (284 from schoolchildren of the public school and 224 from schoolchildren of the private school) were collected and examined. The students from nursery to grade 10 were included in this study. Children who could not obtain their stool themselves and samples contaminated by water, urine otherwise materials were excluded from the study. A short questionnaire was designed which included: (a) demographic data, as age, gender, ethnicity, parent’s occupation (daily basis labor, road cleaners, foreign employers and employees of private firms) and family size; (b) behavioral data, as types of drinking water (government supplied tap water, filtrated tap water in commercial ceramic water filter, commercial mineral water sold in a jar); (c) public or private school. Data were collected by well-trained volunteers.

#### Sample collection

For specimen collection, well labeled, clean, dry, disinfectant free, wide-mouthed plastic containers were distributed to the study population with instruction requesting them to bring about 10-g stool sample the next morning. The containers were labeled with children’s name, code number and date of collection. Stool samples were collected from each student along with demographic and behavioral data using a questionnaire.

#### Macroscopic examination and sample transportation

Collected stool samples were macroscopically examined for color, consistency, and presence of blood, mucus and fragmented or entire helminths, and were immediately fixed with the same volume of 10% formal saline. The fixed stool samples were transported to the Laboratory of (Shi-Gan International College of Science and Technology) SICOST, Kathmandu, Nepal.

#### Microscopic examination

##### Formal-ether sedimentation technique

About 3–4 mL of each formalin-fixed fecal sample were filtered using a cotton gauze in the test tube, mixed (3–4 mL) diethyl ether and vigorously shaken for 5 min, then centrifuged at 3000 rpm for 15 min. The sediment was collected, mounted with iodine solution and microscopically examined for fecal parasites (eggs, oocysts and trophozoites) by using 10× and 40× objectives [13].

##### Data analysis

Pearson’s Chi-Square test value was applied for statistical analysis of results using SPSS 16.0 data analysis software. The odds ratio for risk the factor was calculated using Microsoft Excel. p-values < 0.05 were considered as statistically significant.

### Results

An overall IPIs prevalence of 19.9% (101/508) was found. Multi-parasite infections were detected in 25.7% (26/101) of the stool samples, 22.8% (23/101) was from public school and 2.9% (3/101) from private school (OR: 3.6).

**Fig. 1:**
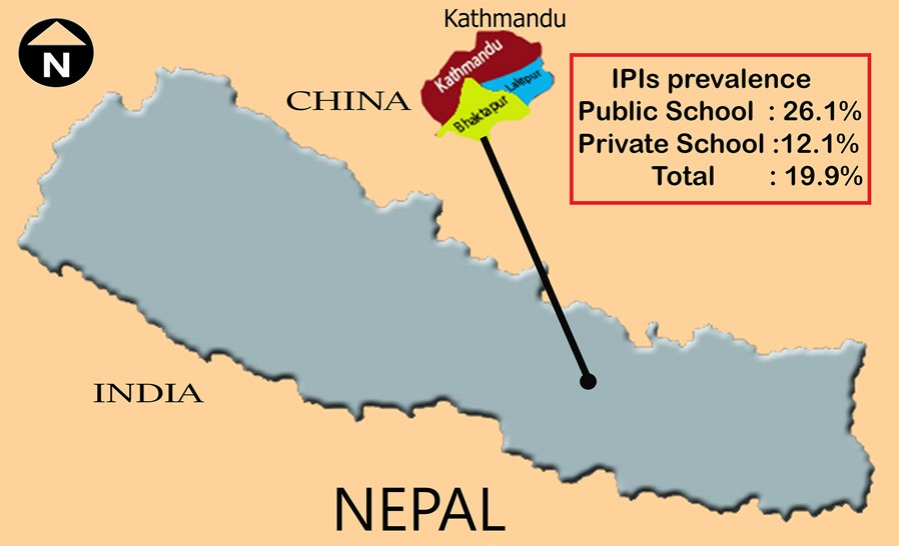
Map of Kathmandu valley of Nepal, showing prevalence of intestinal parasitic infections among schoolchildren. Stool samples were collected from children of public and private school of Kathmandu (capital city of Nepal), examined macroscopically and microscopically by formal ether sedimentation technique (n = 508, M/F; 257/251)

**Fig. 2:**
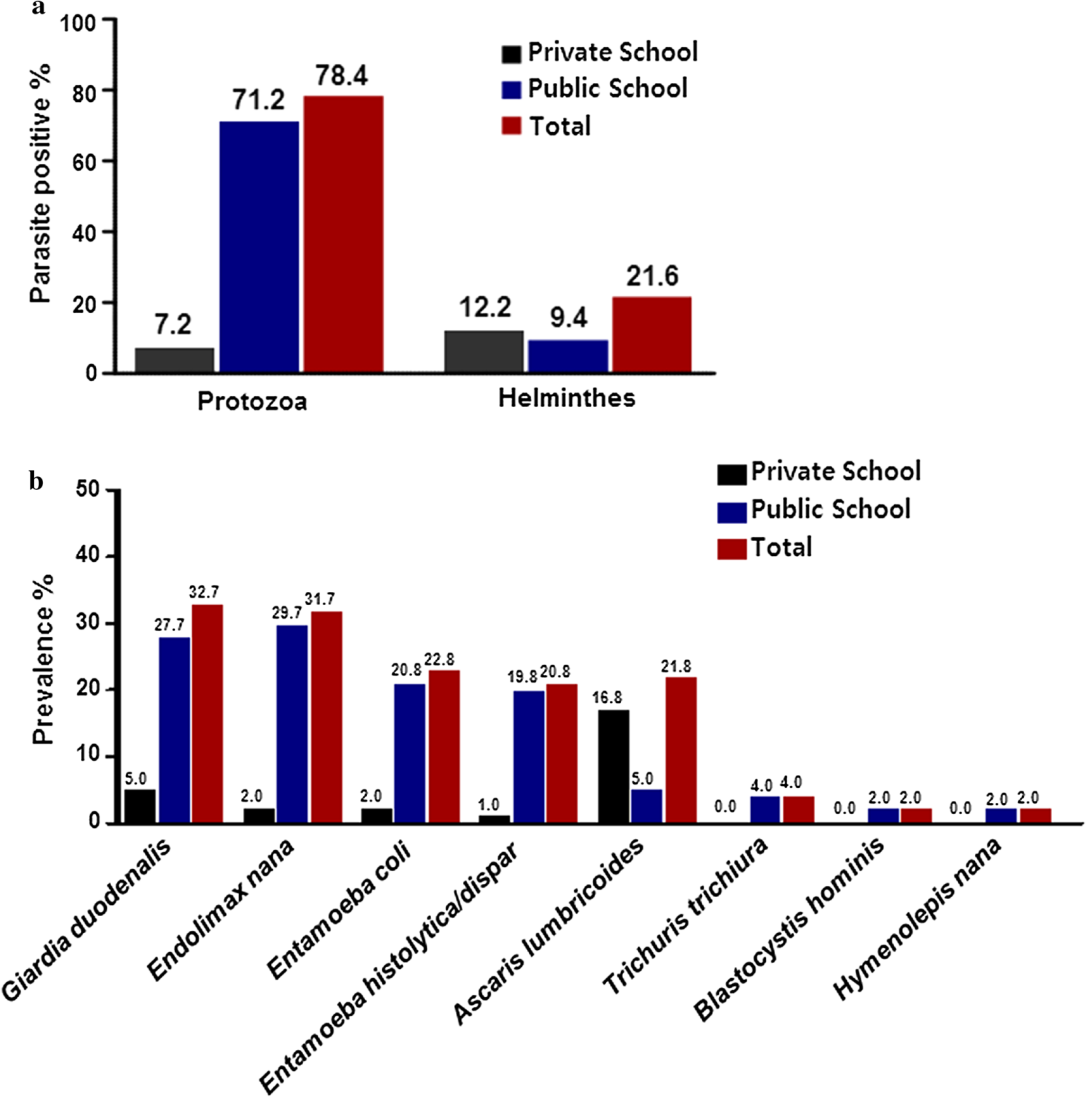
Prevalence of **a** protozoan and helminths intestinal parasites and **b** individual intestinal parasites detected in stool samples of public and private schoolchildren from Kathmandu, Nepal. Stool samples were collected from children of public and private schools of Kathmandu (capital city of Nepal) and examined macroscopically and microscopically by formal ether sedimentation technique (Black: private school; Blue: public school; Red: total) (n = 508, Public/Private; 284/224)

IPIs prevalence was 26.1% (74/284) in public schoolchildren and 12.1% (27/224) in private schoolchildren (p < 0.05) (OR = 2.6) (Fig. 1). According to the gender, a prevalence of 20.0% (50/251) and of 19.8% (51/257) was found among female and male, respectively (p = 0.842) (OR: 1.0) (Table 1). In public school, a prevalence of 26.7% (39/146) and 25.7% (35/138) were found among female and male respectively (OR: 1.1), while in private school female to male prevalence rate were 10.5% (11/105) and 13.4% (16/119) respectively (OR: 0.7). According to the different children age groups, IPIs prevalence rates of 18.5% (43/232) and 21.0% (58/276) were found in 4–10 years old and 11–19 years old group (p = 0.671) (OR: 0.89), respectively. More specifically, in public school, a prevalence of 31.6% (18/57) was found among 4–10 years age group, and 24.7% (56/227) among 11–19 years age group (OR: 1.4), whereas in private school, IPIs prevalence was 14.3% (25/175) in 4–10 years old children and 4.1% (2/49) in 11–19 years old children (OR: 3.91). Intestinal parasites were detected in 22.6% (40/177) population drinking mineral water, followed by 20.0% (14/70) among those drinking filtrated water and by 18.0% (47/261) among those drinking tap water (p > 0.05). Moreover, IPIs prevalence was 19.5% (45/231) in children having a family size less than 5 members, whereas 20.2% (56/277) of children having a family size more than 5 members were positive (OR: 0.95). However, no significant difference was found according to the size of the family (p > 0.05). Based on parent occupation, the prevalence of IPIs in farming family was 29.0% (40/138), followed by 18.8% (22/117) and 14.3% (18/126) among businessman and government job holder (p > 0.05), respectively. Parents of the 16.5% (21/127) positive children were employed in other jobs. According to ethnicity, the prevalence of IPIs among children from the Dalit community 36.2% (17/47) followed by Tibeto-Burman 20.6% (45/218) and Indo-Aryan 16.0% (39/243), (p < 0.05). The prevalence of protozoan infections (78.4%) was approximately thrice the prevalence of helminth infections (21.6%) (Fig. 2a). *Giardia duodenalis* 32.7% (33) was the most prevalent protozoan followed by *Endolimax nana* 31.7% (32), *Entamoeba coli* 22.8% (23), *Entamoeba histolytica/dispar* 20.8% (21) and *Blastocystis hominis* 2.0% (2). Among helminths, *A. lumbricoides* 21.8% (22) was the most prevalent species followed by *Trichuris trichiura* 4.0% (4) and *Hymenolepis nana* 2.0% (2) (Fig. 2b).

**Table 1:** Distribution of IPIs according to socio-demographic characteristics of the schoolchildren in public and private schools of Kathmandu, Nepal

Characteristics	Public school N = 284		Private school N = 224		Total positive (%) N = 101	Total N = 508	p-value	Odds ratio
	Positive (%)	Total	Positive (%)	Total				
Gender								
Male	35 (25.4)	138	16 (13.4)	119	51 (19.8)	257	0.842	1.0
Female	39 (26.7)	146	11 (10.5)	105	50 (20.0)	251		
Age (years)								
4–10	18 (31.6)	57	25 (14.3)	175	43 (18.5)	232	0.671	0.89
11–19	56 (24.7)	227	2 (4.1)	49	58 (21.0)	276		
Source of drinking water								
Mineral water	31 (28.2)	110	9 (13.4)	67	40 (22.6)	177	0.629	
Filtrate water	9 (23.1)	39	5 (16.1)	31	14 (20.0)	70		
Tap water	34 (25.2)	135	13 (10.3)	126	47 (18.0)	261		
Size of family								
≤ 5 member	29 (26.4)	110	16 (13.2)	121	45 (19.5)	231	0.866	0.95
> 5 member	45 (25.1)	174	11 (10.7)	103	56 (20.2)	277		
Occupation of parent’s								
Farming	38 (31.1)	122	2 (12.5)	16	40 (29.0)	138	0.076	
Business	17 (29.3)	58	5 (8.5)	59	22 (18.8)	117		
Others	13 (17.1)	76	8 (15.7)	51	21 (16.5)	127		
Government job	6 (21.4)	28	12 (12.2)	98	18 (14.3)	126		
Ethnicity								
Dalit	11 (44.0)	25	6 (27.3)	22	17 (36.2)	47	0.045	
Tibeto-Burman	35 (27.8)	126	10 (10.9)	92	45 (20.6)	218		
Indo-Aryan	28 (21.1)	133	11 (10.0)	110	39 (16.0)	243

### Discussion

Nepal is a landlocked developing country, located in South Asia where diseases such as diarrhea, gastrointestinal disorders, and intestinal parasites are prevalent [10]. IPIs are considered as the major public health concern worldwide and a significant health concern among schoolchildren of Nepal [14, 15]. Although the prevalence of IPIs in schoolchildren of Nepal is declining [9, 16], in our study 19.9% schoolchildren were found to be affected with IPIs. This prevalence is in agreement with previous reports in Kathmandu 22.0% [11] and Kaski 21.3% [17], which is lower than the reports from other areas of Nepal (31.5–51.9%) [18–21]. This reduced prevalence of IPIs among schoolchildren is due to the periodic deworming and awareness campaigns conducted by the government [22, 23]. Our finding is in agreement with other reports from Nigeria and Nepal showing a higher prevalence of IPIs in public schools (36.7%) and (20.0%) than in private schools (14.1%) and (9.8%), respectively [10, 24]. The instituting of public and private school system in Nepal is based on an unequal socio-economic status of the people [25]. Most of the public schoolchildren belong to the family having a relatively low economic status [19], where they could not offer quality care and good personal hygiene to their children compared to a well-off family. During the study, we found that most of the parents in private school send drinking water in a bottle along with tiffin-box, while public school children drink tap water supplied in the school. Besides that, poor sanitation facility available in the toilet, high exposure to contaminated soil in the playground and low awareness among parents may represent further important reasons for the higher prevalence of IPIs found among public school children in this study.

Similar to previous reports [10, 17], protozoan parasites were found to be prevalent in this study. The higher prevalence of protozoan infections may be owing to higher contamination of drinking water with protozoa compared to helminths. Eight species of parasites were detected in the stool samples. *G. duodenalis* (32.7%) was the most prevalent species, followed by *E. nana* (31.7%) and *E. coli* (22.8%). Various reports from Nepal showed that *G. duodenalis* is a common intestinal parasite with prevalence rates ranging from 13.2 to 73.4% [17, 26–28]. The dominance of protozoa over helminths and the higher prevalence of *G. duodenalis* may depend on its capacity to resist normal level of chlorine treatment in drinking water [29]. A study from Kathmandu reported poor drinking water quality exceeding World Health Organization guideline of coliform count (0 cfu/100 mL) [30].

Alike previous reports [15, 31–33] a high IPIs prevalence among Dalit children (36.2%) was detected in this study, followed by Tibeto-Burman (20.6%) and Indo-Aryan (16.0%). The IPIs prevalence pattern according to ethnicity was similar in both schools. In contrast to our finding, some studies conducted in Kathmandu reported dominant IPIs rate among Tibeto-Burman [32, 33]. The higher prevalence among Dalit and Tibeto-Burman children may be linked with the relatively lower socio-economic status of these ethnic groups [34, 35].

In addition, the prevalence of parasitic infections was statistically independent of gender, source of drinking water and family size. In the public school, most of the children drink water from common water sources whereas in private school, children only share their water bottle. Furthermore, the high susceptibility of parasitic infection among small aged (4–10 years) children reported in this study is in agreement with previous reports from Nigeria [24] and Nepal [36]. This might be attributed to the strengthening of immune status as well as increasing awareness toward hygienic behaviors with age of children. The higher age-wise variance in the prevalence of IPIs in the private school (OR: 3.91) respect to the public school (OR: 1.4), might be due to the early enrollment of children in private schools. Our study has evidenced the relationship between child health status and their parent’s occupation. A high prevalence rate (29%) of IPIs was found in children from farming family, which is concurrent with findings of previous reports [10, 36] from Nepal. This higher prevalence of IPIs in children whose parents adopt farming as the main occupation might be due to their exposure to contaminated soil in farming field, since children help their parents in farming during leisure time. In developing countries like Nepal, the children still get malnourished due to low income, improper nutritional education, inadequate access to health service and lack of safe drinking water and sanitation facilities, and all these factors contribute to the child mortality rate [10]. The higher prevalence rate of IPIs among public schoolchildren can be attributed to their unapproachability to safe drinking water, unhygienic personal habits due to lack of health awareness as most of the children in public school belong to the lower and lower-middle class family. Moreover, the quantity of water supplied in the Kathmandu valley is insufficient relative to its demand. This is also an issue for the sufficiency of sanitation facility in household and schools as well.

### Conclusions

In conclusion, IPIs is an important public health problem among schoolchildren in Kathmandu, Nepal. Our study revealed that the prevalence of IPIs in schoolchildren is significantly associated with types of school. Furthermore, protozoa were found more prevalent than helminths, which might be linked with drinking water. The socioeconomic status and occupation of parents, the age of children and ethnicity are significantly associated with parasitic infections. Health awareness program should provide especially to the parents from the Dalit ethnic group and farmers. Installation of mass water filter and chlorination of drinking water should be applied in both schools. Further studies equipped with advanced microscopic and molecular techniques would be helpful for a proper diagnosis to implement effective prophylaxis.

## Limitations

Due to the limitation of time and resources this study was subjected to incorporate barely only one public and one private school for the comparative study.

## Abbreviations

SICOST: Shi-Gan International College of Science and Technology
WHO: World Health Organization
